# Antimicrobial Efficacy Assessment and Rheological Investigation of Two Different Hand Sanitizers Compared with the Standard Reference WHO Formulation 1

**DOI:** 10.3390/gels9020108

**Published:** 2023-01-27

**Authors:** Sabrina Sommatis, Maria Chiara Capillo, Cristina Maccario, Raffaele Rauso, Edoardo D’Este, Martha Herrera, Mauro Castiglioni, Roberto Mocchi, Nicola Zerbinati

**Affiliations:** 1 UB-CARE S.r.l. Spin-Off, University of Pavia, 27010 Prado, Italy; 2 Head and Neck Unit, Clinica Cobellis, 84078 Vallo della Lucania, Italy; 3 Centro Medico Polispecialistico, 27100 Pavia, Italy; 4 Centro Avanzado de Dermatologia y Laser, San Pedro Sula 21101, Honduras; 5 Lem Compounding Research S.r.l., 20823 Lentate sul Seveso, Italy; 6 Department of Medicine and Surgery, University of Insubria, 21100 Varese, Italy

**Keywords:** alcohol-based hand sanitizer, ABHS, rheology, organoleptic features, antimicrobial activity, MIC, MBC, agar diffusion test, in vitro, in vivo

## Abstract

(1) Background: recently, the use of alcohol-based hand sanitizers (ABHSs) has become very frequent, and an evaluation of the stability and effectiveness of their formulations is a critical topic which should be carefully considered. (2) Methods: starting from the characterization of the hand sanitizers object of the study, our interest was focused on their rheological behavior in order to confirm their intrinsic features, but also the stability of each formulation in different conditions of shear and temperature; the second aspect concerns the antimicrobial assessment through a panel of in vitro and in vivo experimental trials. (3) Results: rheological investigation confirmed good stability for the two hand sanitizers in gel formula with respect to the reference in liquid formula; the antimicrobial activity evaluation showed good efficacy of each formulation both in vitro and in vivo. (4) Conclusions: altogether, our overview presents a valid quality control assessment to ensure the stability and efficacy of an alcohol-based hand sanitizer.

## 1. Introduction

Hospital-acquired infections (HAIs) and community-acquired infections are serious public diseases that are currently the result of prolonged hospitalization or the consequence of common transmission in social environments such as schools, offices or meeting places. The World Health Organization (WHO) and the Centers for Disease Control and Prevention (CDC) propose hand hygiene as the simplest and most preferential control measure in the prevention of transversal microbial or viral transmission [1].

Satisfactory hand cleaning can be achieved by frequent hands washing with water and soap or by alcohol-based hand sanitizers (ABHSs). The WHO definition of hand sanitizers is “An alcohol-containing preparation (liquid, gel, or foam) designed for application to the hands to inactivate microorganisms and/or temporarily suppress their growth” [2]. For a long time, ABHSs have been a useful means to improve sanitary conditions in developing countries.

Recently, during the severe acute respiratory syndrome coronavirus 2 (SARS-CoV-2) pandemic, the habitual consumption of hand sanitizers has exponentially increased globally as a means to reduce and contain horizontal virus transmission. During this emergency state, the WHO published two different hand sanitizer formulations and the relative guidelines for their preparation [3,4]. At the same time, many brands and companies have shifted their core business to hand sanitizer production.

According to the WHO and CDC guidelines, preparations may contain one or more types of alcohol (ethanol, isopropanol, or propanol), other active ingredients with excipients, and humectants. To guarantee the best sanitization, hand sanitizers should contain a recommended content of alcohol (from 60 to 95%) with the function of antimicrobial and antiseptic agents [5]. WHO formulations present some limitations related to their liquid appearance, such as difficulty in spreading or in the delivery of insufficient quantities of the product. Gel hand sanitizer formulations have become popular due to their viscosity, which influences the efficiency, performance, and ease of dispensing, and the use of on-the-go sanitizer to avoid the risk of leakage. Another advantage of the gel formulation is its fast absorption, pleasant smell, and hand feel.

Gel preparations reduce the rate of alcoholic evaporation, so they allow deeper penetration into contaminating organisms and better spread ability [6]. To guarantee these properties, some viscosity enhancer excipients are incorporated in the formulation, such as carbomers, hydroxyethylcellulose (HEC) and xanthan gum (XG) [7]. Carbomers, also called carboxypolymethylene or carbopol (CBP), are cross-linked polyacrylic acid polymers that are suspending agents, stabilizers, and thickeners; in particular, their three-dimensional network is formed by polymeric chain entanglements that, in aqueous solution and upon hydration, start to uncoil and expand [8]. Carbomers can be subdivided into categories characterized by different degrees of cross-linking density. The maximum viscosity degree is reached at a pH of approximately 6.5–7.5, while it decreases at a pH ≥ 9. If a carbomer is added in a formulation that contains alcohol, such as hand sanitizers, the pH must be corrected using neutralizers that do not cause polymer precipitation [6]. HEC is an ether of cellulose that is easily dissolved in water but is insoluble in organic solvents. It is used as a rheological modifier, stabilizer, thickener, suspending agent, and colloidal protective agent. HEC viscosity decreases with increasing alcohol concentration in hand sanitizer [9]. XG is a nontoxic, biocompatible, biodegradable, and inexpensive viscosity enhancer; therefore, in recent years, the field of application of this excipient has increased.

On the other hand, viscous formulations promote dehydration of the skin after prolonged use, obtaining a skin dryness effect [7]. These side effects can be avoided by adding excipients in formulations that balance the side effects with efficacy, safety, and compatibility with different skin types. According to the WHO, fragrances must be limited or completely eliminated within the formulation, as they have been identified as the major cause of contact allergies [2].

Currently, during the SARS-CoV-2 pandemic, ABHSs have been distributed in many countries without an assessment of their effectiveness. In this study, a panel of rheological and microbiological tools were exploited to investigate the efficacy and the chemical-physical stability of two different formulations produced during the emergency state of 2020. In particular, the microbiological trials intend to demonstrate the antimicrobial efficacy of the formulations against the main pathogenic skin strains, demonstrating their sanitizing power. Rheology, on the other hand, is used to study the behavior of viscoelastic parameters when the formulations are subjected to different stress conditions.

The integration of the data obtained could represent a baseline point for a preliminary screening of hand sanitizers efficacy and stability, representing a rapid way to guarantee the safety and the satisfaction of the final consumer. At the same time, it could represent a valid starting point for further investigations that could complete the full characterization of similar formulas.

## 2. Results and Discussion

### 2.1. Organoleptic Characterization

**Figure 1:**
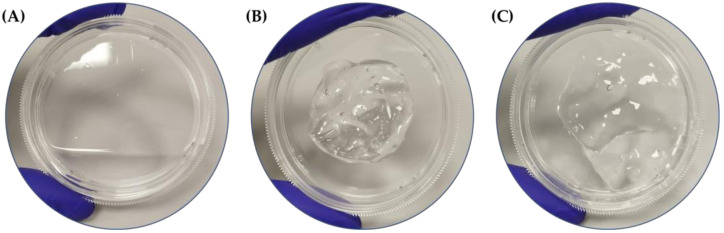
Graphical representation of the appearance of the three formulations object of the study: (**A**) formulation 1 (WHO); (**B**) formulation with carbopol (CBP); (**C**) formulation with hydroxyethylcellulose (HEC) provided by LEM Compounding Research S.r.l.

For a better characterization of the formulations object of the study, a chemical and physical evaluation of these properties that are able to influence customer satisfaction, formulation stability, and efficacy was performed. In order to show the different physical state that determines the appearance of the three formulations, a representative image is shown in Figure 1. The results obtained for appearance, smell, and color (Table 1) are qualitative evaluations of the experimenters involved in the study, while for the pH assessment, data obtained from the mean with the relative standard deviation (SD) of three sample measurements are shown.

### 2.2. Amplitude Sweep Test

**Figure 2:**
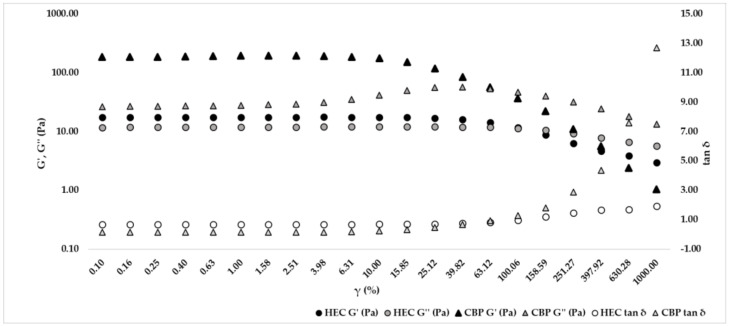
Graphical representation of the results obtained from the amplitude sweep test of formulation with hydroxyethylcellulose (HEC) and formulation with carbopol (CBP) at a temperature of 25 °C and at a frequency value of 1 Hz.

Data obtained with the amplitude sweep test were processed with rSpace for Kinexus software (Malvern Panalytical, Worcestershire, UK). The linear viscoelastic region (LVER) was determined with the sequence (temperature of 25 °C, shear strain 0.1–1000%, and frequency 1 Hz), and in this spectrum, G’ (elastic modulus), G’’ (viscous modulus), and tan δ (tangent of the phase angle) should be constant and run in parallel with increasing shear strain. Data collected are summarized in Table 2 and graphically represented in Figure 2. For the formulation with CBP, G’ was several times larger than G’’, defining a solid-like viscoelastic network of the gel, while for the formulation with HEC, G’ was greater than G’’, confirming a solid-like behavior, but the gap between the two moduli was minor. The crossover point (where G’ = G’’) of the HEC formulation took place at 100.06%, while for the CBP hand sanitizer, it was at approximately 63.12%.

### 2.3. Frequency Sweep Test

**Figure 3:**
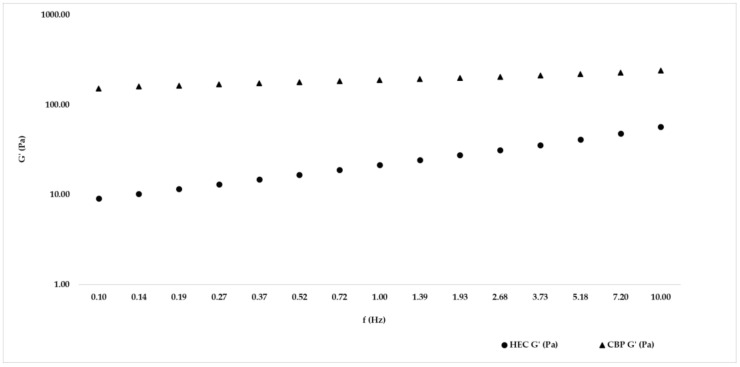
Graphical representation of frequency sweep G’ obtained with the formulation with hydroxyethylcellulose (HEC) and formulation with carbopol (CBP) at a temperature of 25 °C and at a shear strain value of 1%.

To carry out the frequency sweep test, 1% was selected as the fixed shear strain value from the preliminary amplitude sweep test. For hand sanitizers, Figure 3 shows G’ behavior, while Table 3 also includes G’’ and tan δ of both the formulation with hydroxyethylcellulose (HEC) and the formulation with carbopol (CBP), so the prevalence of G’ on G’’ was underlined for both hand sanitizers. For each product, G’ and G’’ increase with increasing frequency. Analyzing the same gel, the G’ modulus was always greater than G’’ within the investigated frequency range, and a solid-like behavior was confirmed because the tan δ value was lower than unity, so the elastic nature of hand sanitizers prevailed in the viscous nature. The formulation with carbopol presented a G’ value greater than the formulation with hydroxyethylcellulose (behaviors shown in Figure 3).

### 2.4. Shear Rate Ramp Test

**Figure 4:**
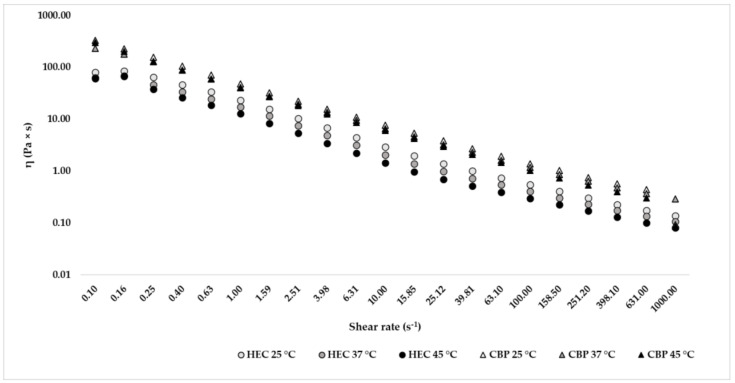
Graphical representation of the viscosity trend of the formulation with hydroxyethylcellulose (HEC) and formulation with carbopol (CBP) at temperatures of 25, 37, and 45 °C with increasing shear rate (s^−1^).

For each hand sanitizer, the results show that shear viscosity (η) decreases as the shear rate increases, demonstrating pseudoplastic behavior for all formulations. As shown in Figure 4, at a low shear rate (0.10 s^−1^), when the formulation was at rest, the products had a high viscosity, which was promoted by the stable network structure formed by the enhancer viscosity excipients. Increasing the shear rate decreased the viscosity because the internal structure became destroyed and deformed, and hand sanitizers became less viscous to allow the product to be spread and improve consumer compliance. Table 4 shows the shear rate to simulate different conditions of the sanitizing gels (rest, extrusion, and application) and the viscosity value obtained for each product [10].

### 2.5. Evaluation of Antimicrobial Activity

Different in vitro tests were performed to better characterize the antimicrobial activity of the three formulations of the study.

#### 2.5.1. Minimum Inhibitory Concentration (MIC) Determination

The data obtained from the evaluation of the MIC by the microtiter plate method are reported in Table 5 and shown for each formulation tested, and the first concentration was able to induce a bacteriostatic effect against the selected microbial strains. The results obtained show a comparable efficacy of the three formulations against the selected strains, except for CBP, which was more effective against *Pseudomonas aeruginosa* than other formulations, and HEC, which was effective at a concentration higher than the others.

#### 2.5.2. Minimum Bactericidal Concentration (MBC) Determination

Starting from the MIC evaluation, the MBC was performed for each formulation against the reference strains to find the first concentration able to reduce the 99.9% of the viable microbial cells. The results obtained are summarized in Table 6 and confirm the MIC efficacy trend except for HEC, which shows a bactericidal concentration equal to the MIC.

#### 2.5.3. Agar Diffusion Test for Antimicrobial Activity Evaluation

The antimicrobial activity of the hand sanitizers was also evaluated using agar disc inhibitory activity against the selected microbial strains *Staphylococcus aureus* (ATCC 6538), *Pseudomonas aeruginosa* (ATCC 9027), *Staphylococcus epidermidis* (ATCC 12228), and *Escherichia coli* (ATCC 87394). The obtained data confirm the antimicrobial activity of the three formulations tested against all strains, and for the interpretation of the results, the inhibitory zones of streptomycin were used as references [11]. The zones of inhibition of the hand sanitizers are shown in Table 7, and the efficacy of each of the three hand sanitizers was evaluated as susceptibility of the microbial strains with respect to each formulation.

#### 2.5.4. In Vivo Efficacy on the Hands of a Cohort of Ten Volunteers

**Figure 5:**
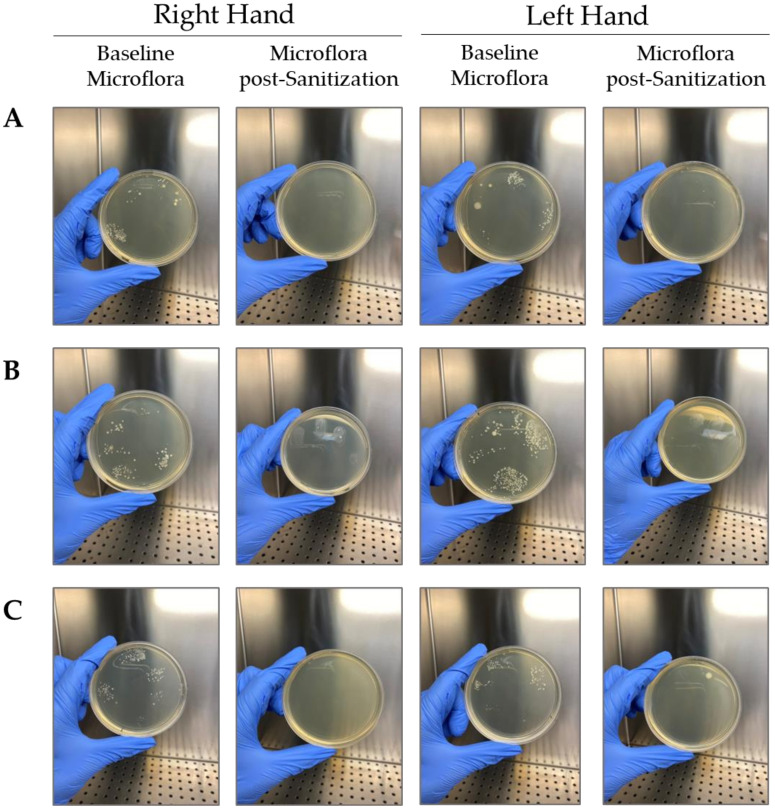
Graphical representation of TSA plates after finger impression in unwashed hands condition (Baseline Microflora) and after clean with the three hand sanitizers object of the study (Microflora post-sanitization): (**A**) formulation with hydroxyethylcellulose (HEC); (**B**) formulation with carbopol (CBP); (**C**) formulation 1—WHO. The picture shows the results obtained for each hand sanitizer on the right and left hand of a subject selected randomly.

To better investigate the antimicrobial activity of the three formulations in the study, a test was conducted on ten volunteers. Table 8 shows the percentage of CFU reduction after treatment with each formulation (formulation 1—WHO, formulation with carbopol—CBP and formulation with hydroxyethylcellulose—HEC) compared to the baseline microflora of each subject involved in the study. Figure 5 shows a graphical representation of the CFU reduction in a representative subject after treatment with the three formulations with respect to the right- and left-hand microflora.

## 3. Conclusions

Alcohol-based hand sanitizers (ABHSs) are formulations commonly used to clean and sanitize hands in the absence of water and soap. Their application is necessary to cope with precarious sanitary conditions in areas lacking adequate water supply and to avoid the transmission of pathogens and infectious agents during the recent severe acute respiratory syndrome coronavirus 2 (SARS-CoV-2) pandemic [3]. The need for large-scale production due to their extensive use led to the development of new formulations by various companies, with the aim of benefitting and sustaining the health-care community. However, most of these products were placed on the market without information about the laboratory-based efficacy evaluation risks when the requirements of stability and efficacy are not satisfied, as they are necessary for the safety and satisfaction of the final consumer.

The disinfectant property of the formulation depends on the concentration and type of alcohol used. Although ethanol may appear to have low bactericidal activity compared to other types of alcohol, it has higher virucidal activity and better skin tolerance. The United States Food and Drug Administration (US FDA), World Health Organization (WHO), and the Centers for Disease Control and Prevention (CDC) have defined ethanol as safe and effective for disinfecting ethanol at concentrations from 60% to 95% (*v*/*v*) [5]. In this study, the experimental design proposed for the quality and efficacy assessment of two different hand sanitizers is presented. The starting point of our overview was the organoleptic definitions of the two formulations containing carbopol (CBP) and hydroxyethylcellulose (HEC) as cross-linker and stabilizer agents, compared with the standard WHO formulation 1. CBP and HEC hand sanitizers have a gel appearance with respect to the WHO-liquid formula thanks to the presence of gelling agents. All formulations present a characteristic ethanol smell and a pH between 6–7; pH plays a key role during gel polymerization, and scientific articles declare that the maximum viscosity degree is reached at a pH of approximately 6.5–7.5 [6]. As previously described, the gel matrix has numerous advantages, such as the reduction of alcohol evaporation, major deeper penetration, and better spread ability.

Rheological characterization was preliminarily carried out for each of the three formulations, but the results obtained for the WHO formulation were not compliant with each other due to the liquid consistency of the formulation and due to the high evaporation of alcohol, the main component of the formulation, caused by the lack of excipients that allowed the creation of a network able to reduce evaporation.

The amplitude sweep test should be conducted in the linear viscoelastic region (LVER), where the viscoelastic shear moduli G’ (elastic modulus) and G” (viscous modulus) of the material are independent of strain and are only related to its molecular structure. In this region, the material structure is maintained intact and the response is independent of the magnitude of the deformation. The point at which the viscoelastic modulus deviates by more than 10% from a constant (plateau) value indicates the critical strain that lead to departure from LVER [12]. Data obtained from this dynamic oscillation test show, for both formulations (CBP and HEC), a G’ greater than G’’, confirming a solid-like viscoelastic network. A difference between the two rheological behaviors is represented by the crossover point at approximately 63% for CBP and 100% for HEC, but also by the gap between G’ and G’’ that is higher for the CBP formulation than the HEC formulation. After the preliminary amplitude sweep test, a frequency sweep test was performed to confirm solid-like behavior for both formulations due to the tan δ value being lower than unity, so the elastic nature of hand sanitizers prevailed in a viscous manner.

The rheological stability was also assessed through an internal shear rate ramp test protocol to simulate different stress conditions to which the formulations can be subjected. The shear rate ramp test determines shear viscosity (η) increasing shear rates, and, based on the behavior, allows a classification of the sample. Fluids can be divided into Newtonian (increasing shear rate viscosity of the sample remains constant) and non-Newtonian fluids subdivided into pseudoplastic fluids (when shear rate increases, the viscosity decreases) and dilatant fluids (with shear rate increasing viscosity increases). Each gel was influenced by a high shear rate by increasing temperature: their viscosity decreased where the temperature increased. At a low shear rate, the temperature did not influence the viscosity of the hand sanitizers. Pseudoplastic behavior is important for a product to spread on the skin to guarantee complete and homogeneous absorption on the skin [13].

For hand sanitizers in gel formula, at low shear rate, simulating the rest in the packaging, a major viscosity is preferred in order to avoid phase separation effect, while at medium-high shear rate values, low viscosity allows for achieving a good level of application and spreading of the hand sanitizer on the human skin in order to guarantee the complete skin covering [14]. The correct viscosity is necessary to allow the spill from the dispenser at a suitable dose. The rheological stability of these formulations is closely linked to their antimicrobial activity, and the lack of bactericidal activity is often due to prolonged storage, which can lead to increased temperature, causing alcohol evaporation and a decrease in effectiveness [15].

After verifying the stability of the formulations object of the study, the evaluation of their antimicrobial efficacy through a panel of microbiological tests was performed. The results show that the growth of selected microbial strains (*Staphylococcus aureus* ATCC 6538, *Pseudomonas aeruginosa* ATCC 9027, *Staphylococcus epidermidis* ATCC 12228, and *Escherichia coli* ATCC 87394) is sensitive to all formulations and decreases with increasing concentrations of each hand sanitizer. This bacteriostatic/bactericidal activity is due to the presence of alcohol as a major component which is able to induce microbial protein denaturation, dissipation of membrane lipids, and consequently disaggregation of membranes [16]. The minimum inhibitory concentration (MIC) investigation demonstrates that *Pseudomonas aeruginosa* is the strain more sensitive to all formulations and, in particular, to the formulation with carbopol (CBP), which shows efficacy at the lower concentration (3.12%). Together with bacteriostatic activity, a bactericidal effect was also found for all hand sanitizers during the in vitro trials, and the trend of the results confirms that of the MICs, with a better minimum bactericidal concentration (MBC) found for the formulation with carbopol (CBP) against *Pseudomonas aeruginosa* (6.25%). Several studies highlight that the effectiveness of alcohol-based hand sanitizers (ABHSs) depends on alcohol concentration, and the final formula with an alcohol content between 60 and 85% is reported to reduce microorganism viability to 99.99% [17]. The sensitivity of the selected strains to the hand sanitizers was confirmed with the agar diffusion test performed according to the Kirby–Bauer method; all strains demonstrated intermediate susceptibility (I, 11 ≥ Ø ≤ 14), except for *Pseudomonas aeruginosa* and *Staphylococcus epidermidis*, which were found to be resistant to the WHO formulation (R, Ø ≤ 10), and *Pseudomonas aeruginosa*, which was sensitive (S, Ø ≥ 15) to the CBP formulation, confirming the previous data and the better efficacy of this formulation compared to the other two.

To better investigate the antimicrobial assessment of hand sanitizers, an in vivo study on ten volunteers was performed, and the results showed comparable efficacy with a microbial reduction of approximately 80% for each of the three formulations. These last data demonstrate an excellent ability to reduce the bacterial load but did not reach a reduction of 99.99%, and none of the three products can be defined as bactericidal in vivo.

In recent years, several studies have been published on the efficacy on the rheological characterization of alcohol-based hand sanitizers, with the final aim of optimizing the formulas by providing greater handleability and hand feel [18,19]. Similarly, this type of formula has been the subject of various efficacy studies to prove the antimicrobial action against the most common pathogenic microflora. The published data on various commercial formulas were comparable with the data obtained in this study for formulas containing an equal ethanol content [20,21]. However, rheological and microbiological tools have not been integrated in order to investigate the stability and efficacy of ethanol-based hand sanitizers, with the ultimate aim of performing a control quality assessment of these formulations.

In conclusion, our overview provides an innovative panel of in vitro and in vivo tests that allow quality control of hand sanitizers in a short time through a good balance between stability, effectiveness, and consequently safety and satisfaction of the final consumer.

## 4. Materials and Methods

### 4.1. Sample Collection

Two different alcohol-based hand sanitizers (ABHSs) were provided by LEM Compounding Research Srl (Lentante sul Seveso, MB, Italy), while the World Health Organization (WHO) formulation 1 was prepared following good manufacturing practices (GMPs) by mixing different ingredients according to the reference standard indications [4,22]. Ingredients for each of the final formulations are described in Table 9.

### 4.2. Organoleptic Properties Evaluation

For a better characterization of the formulations object of the study, a starter assessment of their physical and chemical properties was performed. Appearance, smell, color, and pH are the features investigated as influencing factors for daily use, for its degree of effectiveness, and for the satisfaction of the final customer. Appearance, smell, and odor are physical features defined visually by experimenters involved in the study. Measurement of the pH was performed with a pH meter (1100 L-PH-001, VWR, Milan, Italy) after calibration at 25 °C with two buffer solutions (4.01 and 7).

### 4.3. Amplitude Sweep Test

Hand sanitizer rheological characterization was carried out by Rheometer Kinexus Plus (Malvern Panalytical, Worcestershire, UK), and data processing was performed by rSpace for Kinexus software (Malvern Panalytical, Worcestershire, UK). Measurements were made in triplicate, and fresh samples were loaded. Amplitude sweep tests are usually performed to define the linear viscoelastic region (LVER), where the sample is subjected to a different deformation intensity without breaking its microscopic structure. The LVER is characterized by the linearity of G’ (elastic modulus), G’’ (viscous modulus), and tan δ (tangent phase angle) increasing the shear strain. The rheometer was equipped with cone-plate geometry (CP4/40 SR5476 SS), and analyses were performed under isothermal conditions (25 °C) with a working gap of 0.15 mm. The amplitude sweep sequence was set up at shear strain between 0.1 and 1000% and frequency 1 Hz. An amplitude sweep test was performed as a preliminary test to identify the constant strain value at which to set the frequency sweep test.

### 4.4. Frequency Sweep Test

The frequency was 0.15 mm of the working gap using a cone-plate geometry (CP4/40 SR5476 SS) within the range of 0.1–10 Hz at a shear strain of 1% to avoid changing the microscopic structure of the hand sanitizers remaining in the LVER. By the frequency sweep test, G’, G’’, and tan δ were determined. Tan δ is the result obtained by the ratio G’’/G’, so if the result is more than one, the elastic component is higher than the viscous component; if it is greater than one, the viscous component prevails; in case the ratio is one, the two parameters are equal [23]. The measurements were made in triplicate using fresh samples each time, and data were processed by rSpace for Kinexus software. Data were obtained at 25 °C to study the stability of the formulations at rest or subjected to external frequency during storage.

### 4.5. Shear Rate Ramp Test

The shear rate ramp test was performed with a Kinexus Plus rotational rheometer and processing by rSpace for Kinexus software to investigate the steady shear flow behavior of hand sanitizers at different temperatures (25, 37, and 45 °C) to simulate storage at room temperature and body temperature during application and storage at extreme temperatures. For each sample, 5 min were requested to heat the cartridge and the hand sanitizer. The broad range of shear rates values (0.1–1000 s^−1^ logarithmic scale) was evaluated in order to simulate rest, extrusion, and application of the product [10].

### 4.6. In Vitro Assessment of the Antimicrobial Activity

The antimicrobial activity of the alcohol-based hand sanitizers was evaluated against the most reliable strains of the skin microflora: *Staphylococcus aureus* (ATCC 6538), *Pseudomonas aeruginosa* (ATCC 9027), *Staphylococcus epidermidis* (ATCC 12228), and *Escherichia coli* (ATCC 87394). All strains were purchased by Mecconti S.A.R.L. Sp.z.o.o. (Warszawa, Poland). Bacterial strains were incubated for 24 at 35 ± 2 °C on Tryptic Soy Agar (TSA, Condalab, Madrid, Spain), and after the following subcultures, the third was used to prepare an inoculum in Tryptic Soy Broth (TSB, Condalab, Madrid, Spain) in order to obtain a calibrated culture for the efficacy tests assessment.

#### 4.6.1. Determination of Minimum Inhibitory Concentration (MIC)

MIC is the lowest concentration of a specific antimicrobial needed to prevent the growth of a given antimicrobial substance in vitro (bacteriostatic action). MIC evaluation was performed by using a 96-well microtiter plate method [24]. Serial dilutions of the hand sanitizers in a broth medium suitable for the microbial growth of the selected strains (TSB) were set up according to the following ratio: diluted product 1:2 and subsequent dilutions (range tested from 50% to 0.78%) with respect to the control (broth medium in absence of hand sanitizer). For each dilution, 180 µL was dispensed in duplicate, and 20 µL of the calibrated inoculum (10^6^ CFU/mL final concentration) was added to each well. At the same time, the selected concentrations of the sanitizer diluted in broth without bacteria were dispensed in duplicate and used as background for subsequent optical density (OD) correction readings. The plates were then incubated for 24 h at 37 °C under optimal growth conditions for the selected microbial strains. The same protocol was followed for all selected microbial strains. After 24 h of incubation, spectrophotometric reading was performed at a wavelength of 595 nm using a microplate reader (MultiskanTM Go, Thermo Scientific, Waltham, MA, USA). The value of the corresponding dilution of the product in the absence of bacteria (background) was subtracted from each reading for each concentration tested. Thus, the delta of absorbance was calculated, which measures the OD represented only by the bacterial load present in the well, which is directly proportional to the cells’ viable number.

#### 4.6.2. Determination of Minimum Bactericidal Concentration (MBC)

MBC is represented by the lowest concentration of the product able to reduce 99.9% of cells of a given bacterial strain [17]. Its determination is subsequent to the MIC performance and is based on the preparation of subcultures in agar medium starting from the concentration of the product, which showed no visible growth (MIC). The absence of colonies in subcultures indicates a bactericidal effect. Once the MIC was determined in a 96-well plate, 10 µL of the control and the three increasing concentrations (starting from the MIC) were plated in duplicate on TSA plates incubated for 24 h at 37 °C, the optimal growth conditions for the microbial selected strains. After 24 h of incubation, the qualitative evaluation of presence/absence growth on the plate was performed to register for the hand sanitizer concentration for each strain that was able to induce absence of growth, and thus a bactericidal effect (MBC). The same protocol was followed for all selected microbial strains.

#### 4.6.3. Determination of the Inhibition Zone by Agar Diffusion Test (Kirby–Bauer Method)

An agar diffusion test was performed according to the Kirby–Bauer method to evaluate the test organism susceptibility to the sand sanitizer object of the study. A stock culture in broth medium (TSB) was suitably prepared and incubated for 24 h at 37 °C under optimal growth conditions for each of the selected strains [25]. At the end of the incubation period, a calibrated suspension of 10^7^ CFU/mL was prepared. To obtain a confluent culture, 100 µL of the calibrated inoculum of each strain was inoculated in duplicate on TSA plates. Each plate was divided into three sectors, and a disk of sterile absorbent paper was applied to each of them, containing 100 µL of each condition to be tested [22]. To avoid evaporation and ensure proper diffusion of the product, the plates were left for 3 h at room temperature (RT) and then incubated for 24 h at 37 °C. After 24 h of incubation, the diameters of the inhibition zones of each tested condition were measured and recorded. The diameter of the inhibition zones in millimeters is related to the sensitivity of the bacterium, which is considered sensitive (S), intermediate (I), or resistant (R) according to the criteria established by the guidelines of the Clinical and Laboratory Standards Institute (CLSI) [26]. The results obtained were compared with the standard inhibition parameters of streptomycin, a broad-spectrum antibiotic effective against both gram-positive and gram-negative bacteria [11].

#### 4.6.4. In Vivo Efficacy of Hand Sanitizers

The antimicrobial activity of alcohol-based hand sanitizers was evaluated in vivo in a cohort of ten volunteers aged between 20 and 40 years. The experiments were performed in accordance with relevant named guidelines and regulations and each volunteer was previously informed on the nature and aim of the study. He/she gave a written consent statement and declared not to follow any medical treatment at the time of the study. At the beginning of the trial, the volunteers put their right and left unwashed hands on two TSA plates, pressing and rolling fingers, respectively. TSA agar plates were serially numbered and incubated at 37 °C for 24 h. After incubation, the number of viable colonies enumerated was registered for each plate as baseline microflora for each subject. After the unwashed finger impression, 1 mL of one of the three hand sanitizers was applied to the hands of the subjects. After a homogeneous distribution for at least 20–30 s and complete drying, the finger impression was repeated on two TSA plates (for right and left hand respectively) that were subsequently incubated at 37 °C for 24 h. The following day, the number of viable colonies was enumerated and registered to calculate, for each subject, the efficacy of the hand sanitizer as the ability to reduce the baseline microflora for the right and left hands of each volunteer. The same protocol was followed for each hand sanitizer at a distance of least three hours to obtain a good starter baseline microflora for each trial [25].

## Figures and Tables

**Table 1:** Organoleptic properties and pH of the three formulations object of the study (formulation 1 (WHO); formulation with carbopol (CBP) and formulation with hydroxyethylcellulose (HEC). Values of pH are the results of three independent measurements with relative SD.

Products	Appearance	Smell	Color	pH
WHO	Liquid	Characteristic of ethanol	Transparent	6.77 ± 0.16
CBP	gel	Characteristic of ethanol	Transparent	6.85 ± 0.01
HEC	gel	Characteristic of ethanol	Transparent	7.82 ± 0.19

**Table 2:** Amplitude sweep test data of formulation with hydroxyethylcellulose (HEC) and formulation with carbopol (CBP) at a temperature of 25 °C and at a shear strain value of 1%. The results are presented as averages and relative standard deviations in percentage (RSD %).

Product	G’ (Pa)	G’’ (Pa)	tan δ
HEC	17.42 ± 1.88	11.86 ± 3.79	0.68 ± 3.47
CBP	193.20 ± 0.77	27.82 ± 2.25	0.14 ± 2.54

**Table 3:** Frequency sweep test data obtained for the formulation with hydroxyethylcellulose (HEC) and for the formulation with carbopol (CBP) at a temperature of 25 °C and at a frequency value of 1 Hz. The results are presented as averages and relative standard deviations in percentage (RSD %).

Product	G’ (Pa)	G’’ (Pa)	tan δ
HEC	21.44 ± 6.61	13.90 ± 3.16	0.65 ± 3.57
CBP	188.47 ± 1.14	27.93 ± 1.27	2.3 ± 1.46

**Table 4:** Shear rate ramp test’s results (Pa × s) obtained for the formulation with hydroxyethylcellulose (HEC) and for the formulation with carbopol (CBP) at temperatures of 25, 37, and 45 °C and at different shear rates to simulate different conditions. The results are presented as averages and relative standard deviation percentages (RSD%).

Condition of the Product	Shear Rate (s^−1^)	HEC η 5 °C	CBP η 5 °C	HEC η 7 °C	CBP η 7 °C	HEC η 5 °C	CBP η 45 °C
Rest	10^−2^	80.67 ± 1.25	332.70 ± 5.02	63.75 ± 2.47	231.43 ± 3.44	61.34 ± 5.47	302.67 ± 6.20
Extrusion	10^3^	0.14 ± 3.02	0.09 ± 1.91	0.11 ± 1.85	0.29 ± 3.46	0.08 ± 5.64	0.09 ± 7.15
Application	10^2^	0.55 ± 2.18	1.39 ± 3.09	0.41 ± 1.99	1.18 ± 3.51	0.30 ± 5.64	1.03 ± 6.11

**Table 5:** MIC obtained for the formulation with hydroxyethylcellulose (HEC), for the formulation with carbopol (CBP) and for the formulation 1—WHO against the selected strains *Staphylococcus aureus* (ATCC 6538), *Pseudomonas aeruginosa* (ATCC 9027), *Staphylococcus epidermidis* (ATCC 12228), and *Escherichia coli* (ATCC 87394) (n = 2; r = 2).

Product	*S. aureus*	*P. aeruginosa*	*S. epidermidis*	*E. coli*
WHO	12.5%	6.25%	12.5%	12.5%
CBP	12.5%	3.12%	12.5%	12.5%
HEC	12.5%	6.25%	25%	12.5%

**Table 6:** MBC obtained for the formulation with hydroxyethylcellulose (HEC), for the formulation with Carbopol (CBP), and for formulation 1—WHO against the selected strains *Staphylococcus aureus* (ATCC 6538), *Pseudomonas aeruginosa* (ATCC 9027), *Staphylococcus epidermidis* (ATCC 12228), and *Escherichia coli* (ATCC 87394) (n = 2; r = 2).

Product	*S. aureus*	*P. aeruginosa*	*S. epidermidis*	*E. coli*
WHO	25%	12.5%	25%	25%
CBP	25%	6.25%	25%	25%
HEC	25%	12.5%	25%	25%

**Table 7:** Agar diffusion test for the antimicrobial evaluation of the formulation with hydroxyethylcellulose (HEC), the formulation with carbopol (CBP), and for formulation 1—WHO against the selected strains *Staphylococcus aureus* (ATCC 6538), *Pseudomonas aeruginosa* (ATCC 9027), *Staphylococcus epidermidis* (ATCC 12228) and *Escherichia coli* (ATCC 87394). Data are presented as average and standard deviation (SD) of the diameters (Ø) measured (n = 2; r = 2). Ø ≤ 10 Resistant [R]; 11 ≥ Ø ≤ 14 Intermediate [I]; Ø ≥ 15 Sensitive [S].

Product	*S. aureus* Ø (mm)	*P. aeruginosa* Ø (mm)	*S. epidermidis* Ø (mm)	*E. coli* Ø (mm)
WHO	11 ± 1.4 [I]	10.5 ± 3.5 [R]	10 ± 0.0 [R]	12.5 ± 3.5 [I]
CBP	14 ± 0.0 [I]	15 ± 0.0 [S]	11 ± 0.0 [I]	11.5 ± 0.7 [I]
HEC	13 ± 1.4 [I]	12.5 ± 2.1 [I]	12.5 ± 0.7 [I]	12.5 ± 2.1 [I]

**Table 8:** CFU reduction in percentage (%) post-sanitization with the three formulations with respect to the baseline microflora of ten volunteers. For each subject, the CFU reduction (%) after treatment with the different hand sanitizers and the mean ± SD obtained from the data collected of the right and left hand of each volunteer for each formulation are reported.

Volunteers	HEC CFU Reduction (%)		CBP CFU Reduction (%)		WHO CFU Reduction (%)	
	RX	LX	RX	LX	RX	LX
V1	91.76	81.69	86.40	66.67	86.96	98.04
V2	55.84	89.74	59.32	97.10	50.06	51.85
V3	50.00	85.50	68.09	66.67	92.67	94.75
V4	93.67	97.83	93.75	95.38	99.32	76.62
V5	78.00	89.33	75.70	35.82	98.00	93.00
V6	98.44	98.02	97.49	99.59	99.00	99.50
V7	96.43	99.00	98.65	18.46	94.55	95.83
V8	75.20	75.33	93.53	58.43	89.83	39.39
V9	97.00	90.00	99.61	98.70	50.00	59.17
V10	87.43	83.05	99.00	97.54	99.67	95.00
mean ± SD	85.66 ± 13.57		80.29 ± 23.54		83.16 ± 20.54	

**Table 9:** Formulations collected for the study design with relative composition. Formulation 1—WHO; Formulation with Carbopol—CBP; Formulation with Hydroxyethylcellulole—HEC.

Formulation	Ingredients
WHO	Ethanol, Hydrogen Peroxide, Glycerol, Aqua
CBP	Aqua, Alcohol Denat, Butylene Glycol, Acrylates/c10-30 Alkyl Acrylate Crosspolymer, Xanthan Gum, Aminomethyl Propanol
HEC	Aqua, Alcohol Denat, Hydroxyethylcellulose

## Data Availability

Data are included in the text; raw data are available from the corresponding author upon request.
